# Transcriptional response modules characterize IL-1β and IL-6 activity in COVID-19

**DOI:** 10.1016/j.isci.2020.101896

**Published:** 2020-12-07

**Authors:** Lucy C.K. Bell, Cem Meydan, Jacob Kim, Jonathan Foox, Daniel Butler, Christopher E. Mason, Sagi D. Shapira, Mahdad Noursadeghi, Gabriele Pollara

**Affiliations:** 1 Division of Infection & Immunity, University College London, Cruciform Building, Gower Street, London WC1E 6BT, UK; 2 Hospital for Tropical Diseases, University College London Hospitals NHS Trust, London, UK; 3 The HRH Prince Alwaleed Bin Talal Bin Abdulaziz Alsaud Institute for Computational Biomedicine, Weill Cornell Medicine, New York, NY, USA; 4 Herbert Irving Comprehensive Cancer Center, Columbia University, New York, NY, USA; 5 Department of Systems Biology, Columbia University, New York, NY, USA; 6 Department of Physiology and Biophysics, Weill Cornell Medicine, New York, NY, USA; 7 The WorldQuant Initiative for Quantitative Prediction, Weill Cornell Medicine, New York, NY, USA; 8 The Feil Family Brain and Mind Research Institute, Weill Cornell Medicine, New York, NY, USA; 9 Department of Infection, Royal Free London NHS Trust, London, UK

**Keywords:** Immunology, Virology, Transcriptomics

## Abstract

Dysregulated IL-1β and IL-6 responses have been implicated in the pathogenesis of severe Coronavirus Disease 2019 (COVID-19). Innovative approaches for evaluating the biological activity of these cytokines *in vivo* are urgently needed to complement clinical trials of therapeutic targeting of IL-1β and IL-6 in COVID-19. We show that the expression of IL-1β or IL-6 inducible transcriptional signatures (modules) reflects the bioactivity of these cytokines in immunopathology modelled by juvenile idiopathic arthritis (JIA) and rheumatoid arthritis. In COVID-19, elevated expression of IL-1β and IL-6 response modules, but not the cytokine transcripts themselves, is a feature of infection in the nasopharynx and blood but is not associated with severity of COVID-19 disease, length of stay, or mortality. We propose that IL-1β and IL-6 transcriptional response modules provide a dynamic readout of functional cytokine activity *in vivo*, aiding quantification of the biological effects of immunomodulatory therapies in COVID-19.

## Highlights

• Transcriptional response modules reflect IL-1β and IL-6 activity *in vivo*
• Response modules are superior to single gene transcripts in measuring cytokine activity
• Elevated IL-1β and IL-6 activity is a feature of COVID-19 disease in blood and tissues
• COVID-19 disease severity is not associated with greater IL-1β or IL-6 activity

## Graphical Abstract

**Figure:**
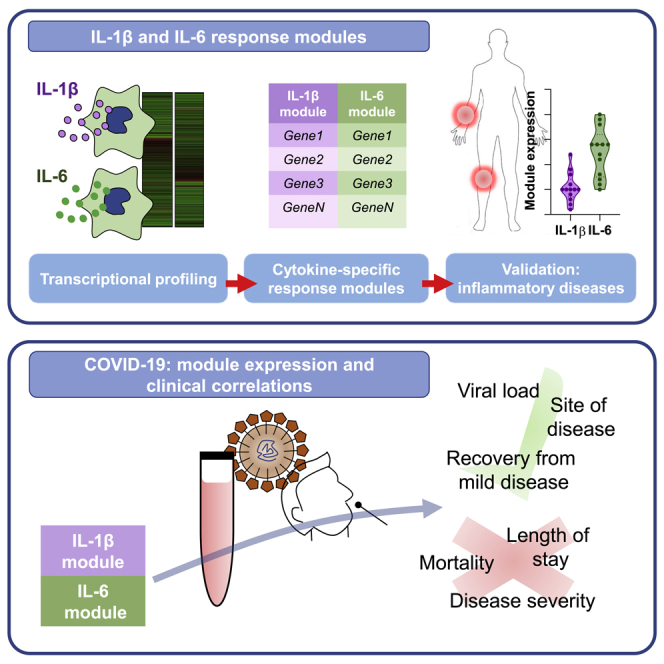

## Introduction

Severe Coronavirus Disease 2019 (COVID-19) typically occurs over a week from symptom onset, when viral titers have diminished, suggesting a dysregulated host inflammatory response may be driving the pathogenesis of severe disease (Bullard et al., 2020; Huang et al., 2020; McGonagle et al., 2020). Elevated IL-1β and IL-6 responses have each been associated with disease severity (Huang et al., 2020; Liao et al., 2020; Qin et al., 2020; Ravindra et al., 2020; Zhang et al., 2020; Zhou et al., 2020b). In addition, the hyperinflammatory state in COVID-19 is reported to resemble some aspects of hemophagocytic lymphohistiocytosis (HLH), a condition that may benefit from therapeutic IL-1β blockade (Mehta et al., 2020). These observations have generated hypotheses that IL-1β and/or IL-6 may be key drivers of pathology in severe COVID-19 and led to clinical trials of IL-1β and IL-6 antagonists in this context (Maes et al., 2020). Randomized studies to date investigating the role of tocilizumab, a humanized monoclonal antibody against the IL-6 receptor, have shown no clinical benefit, but immunophenotyping beyond the measurement of single cytokines, before or after drug administration, was not recorded or correlated with clinical responses at the individual patient level (Hermine et al., 2020; Salvarani et al., 2020; Stone et al., 2020).

The measurement of individual cytokines at the protein or RNA level may not reflect their biological activity accurately within multivariate immune systems that incorporate redundancy and feedback loops. To address this limitation, we have previously derived and validated gene expression signatures, or modules, representing the transcriptional response to cytokine stimulation, using them to measure functional cytokine activity within genome-wide transcriptomic data from clinical samples (Bell et al., 2016; Byng-Maddick et al., 2017; Dheda et al., 2019; Pollara et al., 2017, 2019). However, transcriptional modules to quantify IL-1β or IL-6 response have not been used in COVID-19 to quantify the bioactivity of these cytokine pathways *in vivo*. In the present study, we have sought to address this gap, describing the derivation and validation of IL-1β and IL-6 inducible transcriptional modules and testing the hypothesis that these modules can be used in the molecular assessment of the pathophysiology and the response to therapeutic cytokine blockade of inflammatory conditions, including COVID-19.

## Results

### Identification and validation of IL-1β and IL-6 transcriptional modules

**Figure 1:**
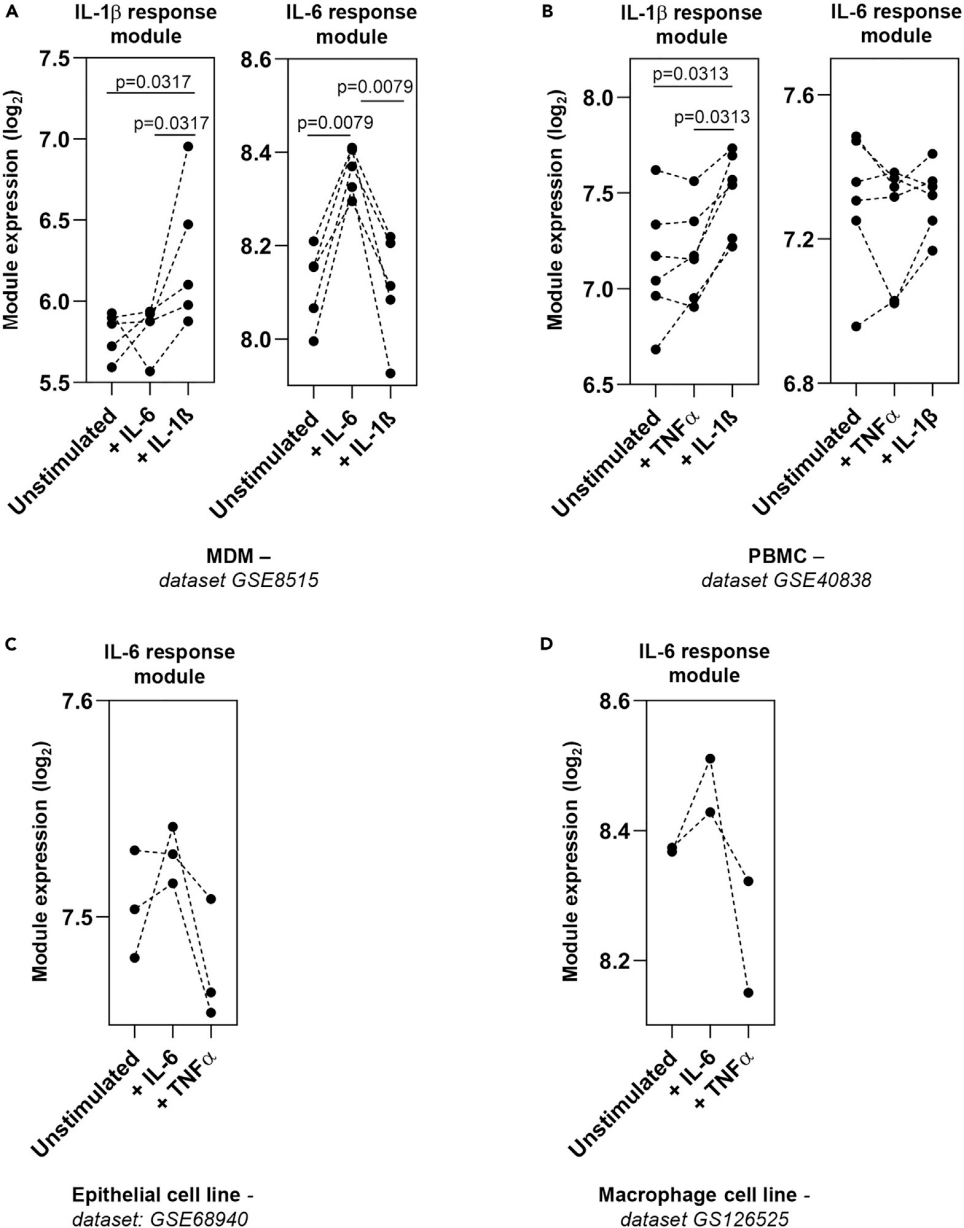
Validation of cytokine response modules (A–D) Geometric mean module expression in (A) MDM stimulated *in vitro* with either IL-1β (15 ng/mL) or IL-6 (25 ng/mL) for 4 h (Jura et al., 2008), (B) PBMC stimulated with TNFα (20 ng/mL) or IL-1β (10 ng/mL) for 6 h (Boisson et al., 2012), (C) human renal proximal tubular epithelial (HK-2) cells stimulated with IL-6 (200 ng/mL) or TNFα (100 ng/mL) for 1.5 h (O'Brown et al., 2015), and (D) human macrophage cell lines (THP-1) stimulated with IL-6 (50 ng/mL) or TNFα (10 ng/mL) for 2 h (Das et al., 2020). Transcriptomic datasets are designated adjacent to figure panels. p values derived from two-tailed Mann-Whitney test.

We first sought to derive transcriptional modules that identified and discriminated between the response to IL-1β and IL-6 stimulation. We have previously derived an IL-1β response module from cytokine-stimulated fibroblasts (Table S2) (Pollara et al., 2019). As in our prior studies (Bell et al., 2016; Pollara et al., 2017, 2019), we used the geometric mean of the constituent genes in a module as a summary statistic to describe the relative expression of the module. We demonstrate that in both monocyte-derived macrophages (MDM) and peripheral blood mononuclear cells (PBMC) (Boisson et al., 2012; Jura et al., 2008), IL-1β stimulation induced greater expression of the IL-1β response module than either IL-6 or TNFα stimulation, where there was no increased expression above unstimulated cells (Figures 1A and 1B). To identify an IL-6 response module that was able to discriminate from the effects of IL-1β, we identified one study that had stimulated human MDM with either IL-1β (15 ng/mL) or IL-6 (25 ng/mL) for 4 h (Jura et al., 2008). Hierarchical clustering identified genes induced by IL-6 but not IL-1β, and we termed this the IL-6 response module (Table S2). Internal validation of this module confirmed increased expression in IL-6 stimulated MDM (Figure 1A). Testing the IL-6 module in other datasets demonstrated elevated expression following IL-6, but not TNFα, stimulation of human kidney epithelial and macrophage cell lines (Das et al., 2020; O'Brown et al., 2015) (Figures 1C and 1D), whereas no elevated expression of the IL-6 module was observed following IL-1β or TNFα stimulation of MDM or PBMC (Figures 1A and 1B). These findings demonstrated that the IL-1β and IL-6 response modules could detect the effects of their cognate cytokines and discriminate these from each other and from an alternative inflammatory cytokine stimulus, TNFα.

### IL-1β and IL-6 module expression in chronic inflammation

**Figure 2:**
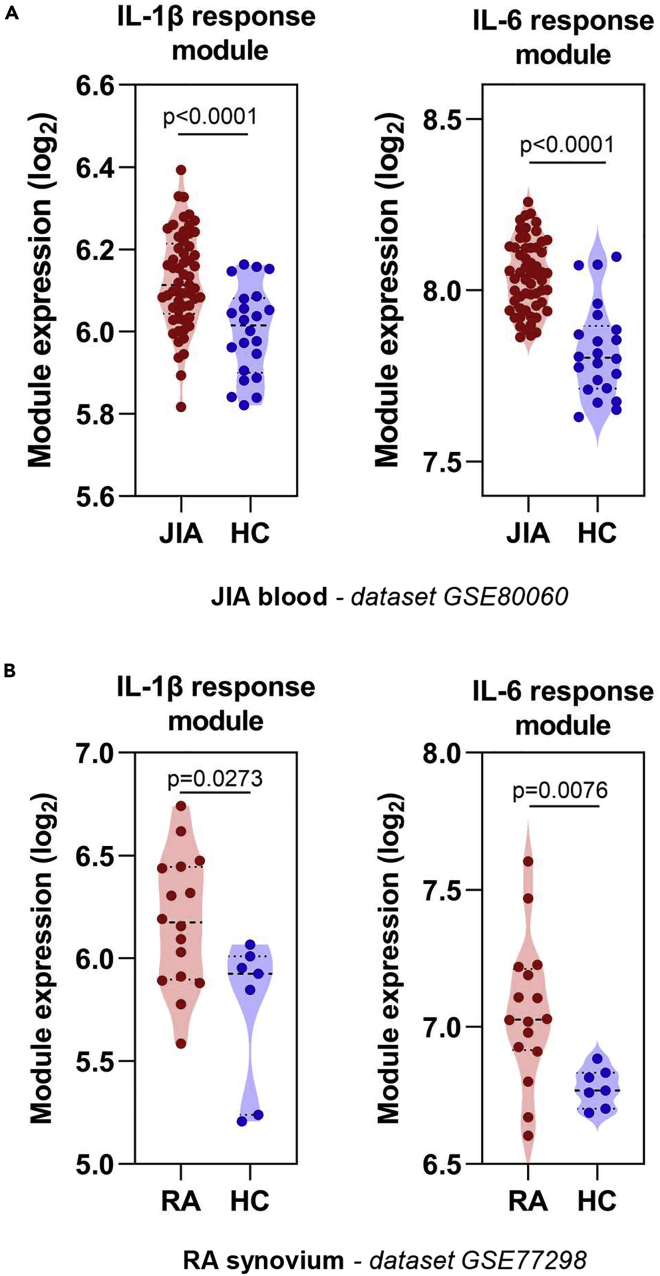
Cytokine response module expression in chronic inflammatory conditions (A and B) Geometric mean expression of IL-1β and IL-6 cytokine response modules in (A) blood of patients with JIA compared with healthy controls (Brachat et al., 2017) and (B) in the synovium of RA patients compared with that of healthy controls (Broeren et al., 2016). Transcriptomic datasets are designated adjacent to figure panels. p values derived from two-tailed Mann-Whitney test.

To determine whether IL-1β and IL-6 response modules were able to detect elevated cytokine bioactivity *in vivo*, we assessed the blood transcriptome of juvenile idiopathic arthritis (JIA) and rheumatoid arthritis (RA) patients. These are conditions in which elevated IL-1β and IL-6 activity are considered to play a key role in disease pathogenesis, evidenced by clinical improvement following therapeutic antagonism of these cytokines (De Benedetti et al., 2012; Fleischmann, 2017; Nikfar et al., 2018; Ruperto et al., 2012). The blood transcriptome of untreated JIA patients displayed elevated IL-1β and IL-6 bioactivity (Figure 2A) (Brachat et al., 2017), but this was not consistently evident in several RA blood transcriptome datasets (Figure S1) (Lee et al., 2020; Macías-Segura et al., 2018; Tasaki et al., 2018). Discrepancies between molecular changes in blood and tissues have been previously described in RA (Lee et al., 2020), and therefore we tested the hypothesis that, in contrast to blood, elevated IL-1β and IL-6 bioactivity was a feature of the synovium in RA. Consistent with this hypothesis, a separate transcriptomic dataset of synovial membrane biopsies from patients with RA (Broeren et al., 2016) showed elevated levels of both IL-1β and IL-6 response module expression compared with non-RA synovium (Figure 2B).

**Figure 3:**
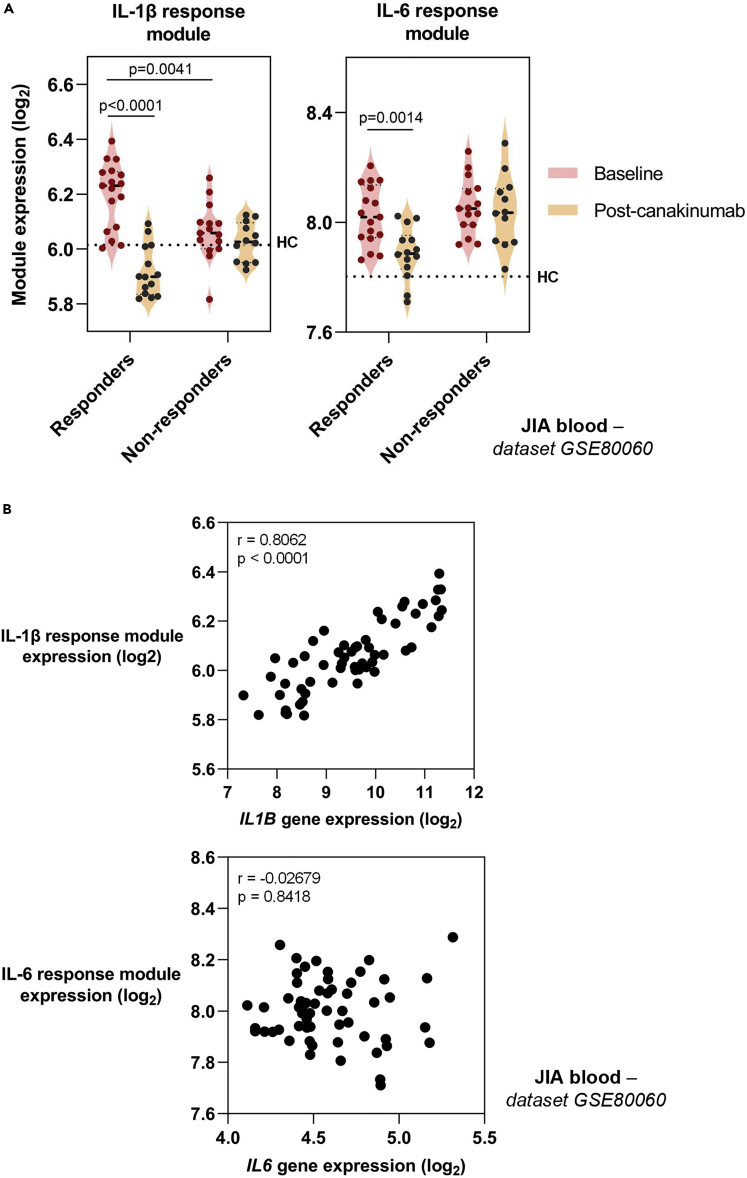
Effect of canakinumab on expression of cytokine response modules and genes (A) Geometric mean expression of IL-1β and IL-6 cytokine response modules in JIA patients before and 3 days after administration of canakinumab (Brachat et al., 2017). Patients were subdivided into good responders (90%–100% improvement) and non-responders (0%–30% improvement). Dotted lines indicate median module or gene expression in healthy controls (HC) population in same dataset. p values derived from two-tailed Mann-Whitney test. (B) Relationship between expression of cytokine response modules and cytokine genes. Statistical assessment of correlation made by Spearman Rank correlation. r = correlation coefficient. Transcriptomic dataset designated adjacent to figure panels.

We used the elevated cytokine activity in the blood of JIA patients to test the hypothesis that therapeutic cytokine modulation would result in changes in cytokine bioactivity as determined by module expression. We made use of the blood transcriptome of JIA patients 3 days following administration of canakinumab, a human monoclonal antibody to IL-1β (Brachat et al., 2017). Patients who had a therapeutic response to canakinumab showed elevated IL-1β module expression, which reduced 3 days after canakinumab administration (Figure 3A). In contrast, in those who had no treatment response, IL-1β module expression was lower at baseline and was unaffected by canakinumab (Figure 3A). Unlike the differences seen in the IL-1β module between responders and non-responders, there were no differences between these groups in IL-6 module expression at baseline (Figure 3B). This indicated that these two cytokine response modules quantified two distinct biological processes. Interestingly, expression of the IL-6 module was also diminished after canakinumab treatment in patients who responded to treatment, suggesting that IL-6 activity may be downstream of IL-1β in this context. Of note in these populations, the expression of the *IL1B* gene correlated with that of the IL-1β response module, but the same was not evident between IL-6 module and *IL6* gene expression (Figure 3C), illustrating an example in which cytokine gene expression itself may not necessarily reflect the functional activity of that cytokine.

### IL-1β and IL-6 bioactivity in COVID-19

**Figure 4:**
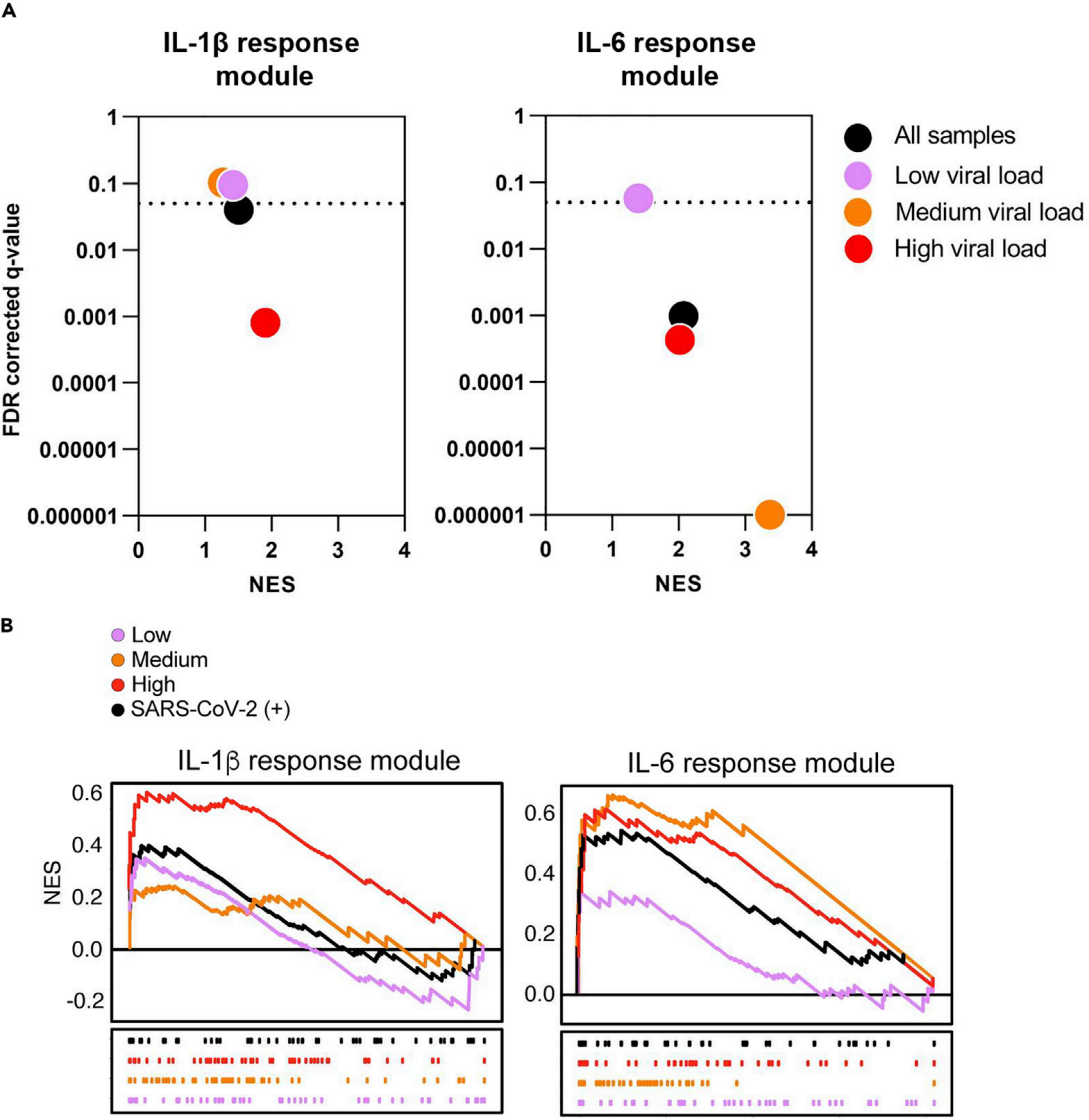
Cytokine response modules at the site of disease in COVID-19 (A) Gene set enrichment analysis (GSEA) of the IL-1β and IL-6 modules was applied to nasopharyngeal swabs from SARS-CoV-2-infected and uninfected individuals. Patients were stratified into low (pink), medium (orange), and high (red) viral loads as previously described (Ramlall et al., 2020). GSEA was used to determine the level of engagement for the respective modules in the context of SARS-CoV-2 infection (Subramanian et al., 2005), in line with previously published analysis of this dataset (Ramlall et al., 2020). Normalized enrichment scores (NES) are shown on the x axes, and measurement of statistical significance (false detection rate q-value) is shown on the y axes. The threshold for significance (q = 0.05) is shown by the dotted lines; data points below the dotted lines are significantly enriched for the relevant module in each group of SARS-CoV-2 positive patients, in comparison to the control group. (B) Leading edge enrichment plots from GSEA of the cytokine modules for each comparison.

We tested the hypothesis that elevated IL-1β and IL-6 bioactivity is a feature of COVID-19 disease. We initially explored the induction of IL-1β and IL-6 activity at the site of COVID-19 disease, by profiling transcriptional responses in nasopharyngeal swabs from 495 control and 155 SARS-CoV-2-infected individuals (Butler et al., 2020; Ramlall et al., 2020). Gene set enrichment analysis (GSEA) was used as an alternate method of module enrichment scoring (Subramanian et al., 2005), in line with previous analyses of this dataset (Ramlall et al., 2020). Although the IL-1β response module was modestly induced by SARS-CoV-2 infection, the IL-6 response module was significantly enriched in transcriptional programs induced by this viral infection (Figure 4). Moreover, we found that SARS-CoV-2 viral loads were positively associated with cytokine activity, with enrichment of IL-1β and IL-6 responses observed in individuals with the upper tertile of measured viral loads, whereas patients with the lowest tertile viral titers did not show induction of responses to either cytokine (Figure 4). The greatest IL-6 responses were in fact observed in individuals with intermediate viral titers, in whom significant induction of IL-1β activity was not seen (Figure 4). Together, these findings suggest that both IL-1β and IL-6 activity are a feature of the host response at the site of SARS-CoV-2 infection and are likely to be driven by increasing viral replication *in vivo*.

**Figure 5:**
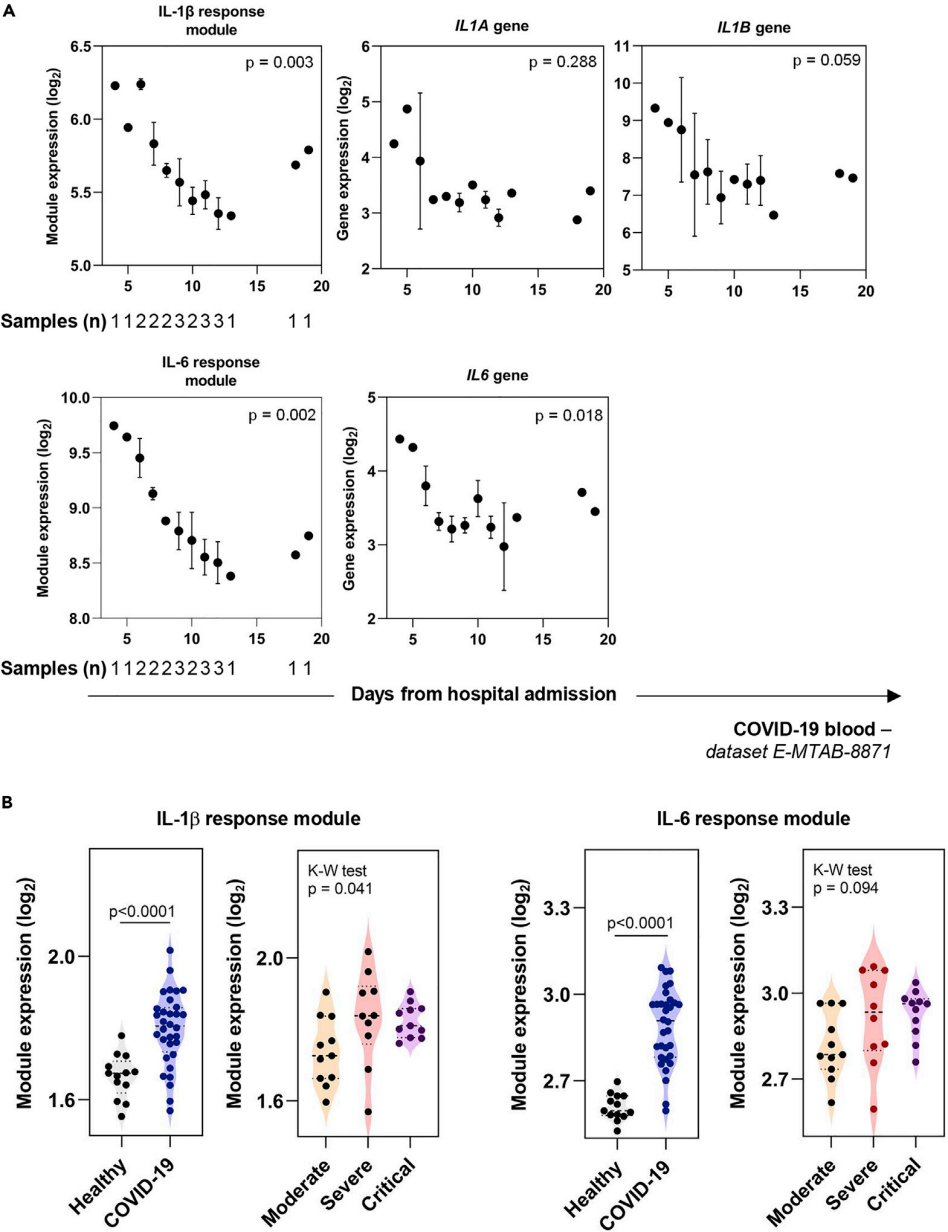
Cytokine response module and gene expression in COVID-19 blood samples (A) Geometric mean expression of IL-1β and IL-6 response module and *IL1A*, *IL1B*, and *IL6* gene expression in patients admitted with COVID-19 (Ong et al., 2020). Number of patient samples at each time point designated on first plot of each row but applicable for all panels. Where more than one sample was available at any time point, the mean expression +/− SEM is plotted. Kruskal-Wallis test was performed on binned time points 4–6, 7–9, 10–12, and 12 + days following hospitalization, corresponding to 4, 7, 8, and 3 samples in each of these categories. The p values shown represent Kruskal-Wallis tests with time since hospital admission as the independent variable, where a threshold of 0.01 (corrected for multiple testing by the Bonferroni method) is required for a single test to be classed as significant (significant p values indicated in bold text). (B) Geometric mean expression of IL-1β and IL-6 response modules in whole blood transcriptomic profiles from patients admitted with moderate (n = 11), severe (n = 10), or critical (n = 11) COVID-19, in comparison to healthy controls (n = 13) (Hadjadj et al., 2020). In this study, samples were collected from patients at the time of admission to hospital, a median of 10 days (IQR 9–11 days) from symptom onset. A Mann-Whitney test was used to assess differences in module expression between all COVID-19 patients and healthy controls (p value on figure), and a Kruskal-Wallis test was used to determine variability in module expression between the grades of COVID-19 disease severity.

As clinical deterioration in COVID-19 occurs after peak viral replication in the airways has subsided, we tested the hypothesis that IL-1β and IL-6 activity was also related to disease severity. We initially explored IL-1β and IL-6 activity in the blood of three patients with mild-moderate COVID-19 disease who were admitted to hospital and recovered (Ong et al., 2020). This dataset was generated using the Nanostring system and consisted of 579 mRNA targets, which included only 7/57 (12.2%) and 7/41 (17.1%) constituent genes of the IL-1β and IL-6 response modules, respectively (Table S2). We demonstrated that IL-1β and IL-6 submodules, generated from these shorter lists of constituent genes, were still able to recapitulate all the findings from Figure 3 (Figure S2). The expression of these submodules in the blood transcriptome of this small number of COVID-19 patients revealed variation in IL-1β and IL-6 bioactivity over the period of hospitalization, with higher expression seen earlier during hospital admission and a reduction as patients recovered (Figure 5A). This time-associated relationship with clinical recovery was not seen for the expression of the *IL1A*, *IL1B*, and *IL6* genes (Figure 5A). We extended these analyses by assessing the transcriptome of blood samples collected at the time of hospital admission from 32 COVID-19 patients presenting with varying levels of disease severity (Hadjadj et al., 2020). These data, also collected using the Nanostring system, revealed expression of the IL-1β and IL-6 cytokine submodules was clearly elevated in COVID-19 compared with healthy controls (Figure 5B). However, strikingly, there was only minimal variability in IL-1β and no variability in IL-6 submodule expression between the different levels of COVID-19 disease severity (Figure 5B).

**Figure 6:**
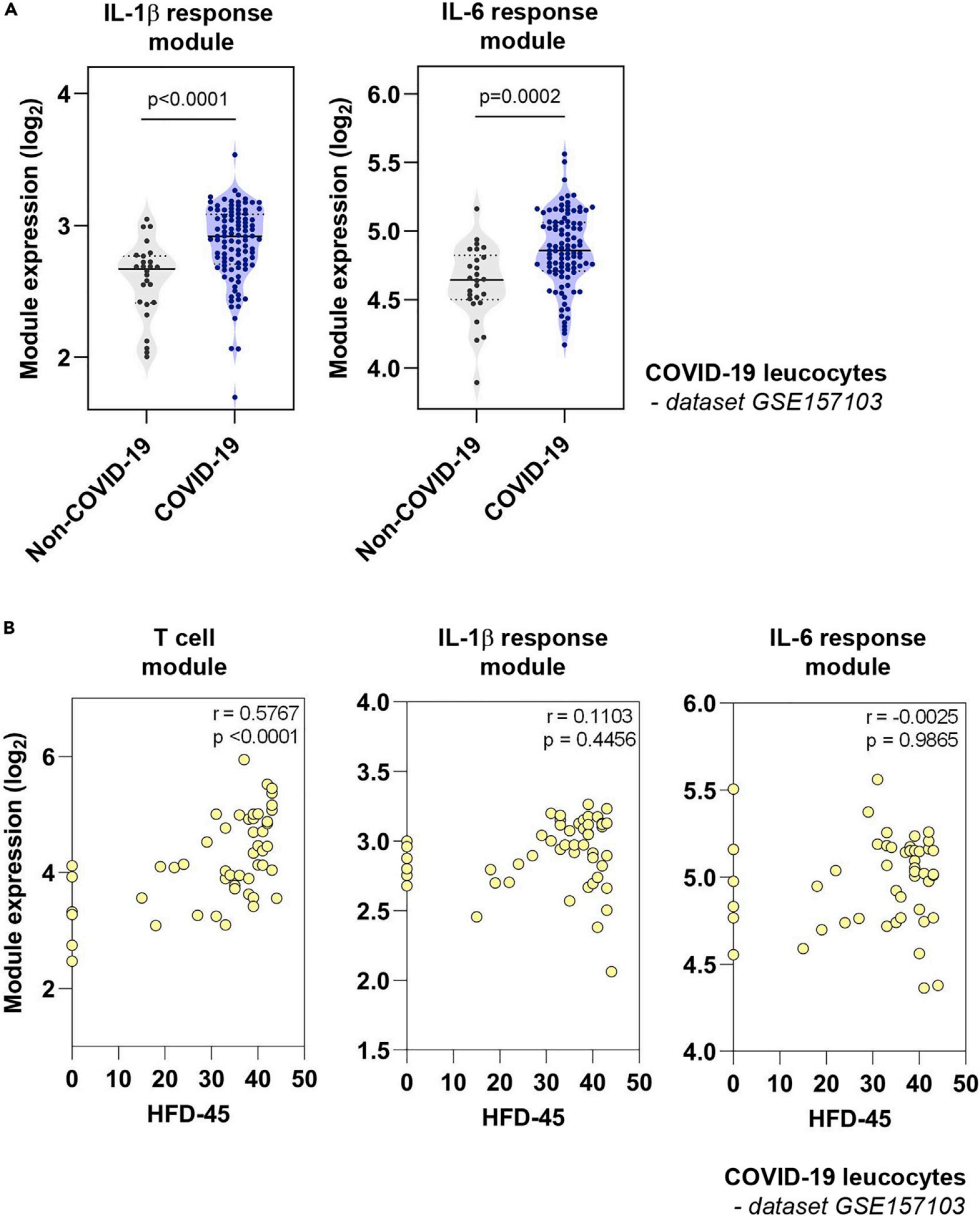
Relationship between cytokine response module expression at admission in COVID-19 and clinical outcome (A) Geometric mean expression of IL-1β and IL-6 response modules in transcriptomic profiles of blood leukocytes collected from 101 COVID-19 and 24 non-COVID-19 patients. In this study, samples were collected from patients at a median of 3.37 days from admission to hospital (Overmyer et al., 2020). p values derived from two-tailed Mann-Whitney test. (B) In patients from this cohort who were not admitted to ITU, the relationship between expression of cytokine response modules, or a previously validated T cell module (Pollara et al., 2017), and the number of hospital-free days at day 45 (HFD-45) following hospital admission (whereby zero days indicated continued admission or death) is shown. Statistical assessment of correlation made by Spearman Rank correlation. r = correlation coefficient.

Finally, we tested the hypothesis that elevated IL-1β and IL-6 transcriptional activity in blood could predict clinical outcome in COVID-19. We assessed the transcriptome of blood leukocytes from 101 COVID-19 and 24 non-COVID-19 patients admitted to hospital (Overmyer et al., 2020). As seen in the whole blood transcriptome analysis (Figure 5), leukocytes from COVID-19 patients also demonstrated elevated IL-1β and IL-6 module activity compared with controls (Figure 6A), and once again this distinction was not seen in IL1A, IL1B, and IL6 gene expression (Figure S3). Clinical outcome in this cohort was determined from the number of hospital free days at day 45 (HFD-45) following hospital admission, whereby zero days indicated continued admission or death (Overmyer et al., 2020). Prognostication models have identified decreased lymphocyte counts as predictors of clinical deterioration (Gupta et al., 2020). Focusing on COVID-19 patients not requiring ICU admission, we reproduced this observation, demonstrating a positive correlation between HFD-45 and the expression of a transcriptional module that reflects T cell frequency *in vivo* (Pollara et al., 2017) (Figure 6B). In contrast, neither IL-1β nor IL-6 response module expression at the time of study recruitment was associated with HFD-45, indicating that, in this dataset, transcriptional activity of these cytokines was not predictive of clinical outcome from COVID-19 infection (Figure 6B).

## Discussion

The protracted clinical course, inverse relationship between viral load and symptom progression, and the association between inflammation and worse clinical outcomes support a hypothesis whereby severe COVID-19 disease is predominantly driven by an exaggerated inflammatory response (Bullard et al., 2020; Huang et al., 2020). Both IL-1β and IL-6 may play a role in this process (Huang et al., 2020; Liao et al., 2020; Qin et al., 2020; Ravindra et al., 2020; Zhang et al., 2020; Zhou et al., 2020a), and cytokine-modulating therapies are now being tested in COVID-19 clinical trials. In this study we utilized transcriptional modules derived from cytokine-stimulated cells to demonstrate that their expression, but not that of their cognate cytokine genes, provided a quantitative readout for cytokine bioactivity *in vivo*, both in the context of COVID-19 and chronic inflammatory conditions.

We show that in COVID-19, IL-1β and IL-6 cytokine activity is detectable at a site of disease, the nasopharynx, where greater IL-6 bioactivity in particular is associated with higher levels of SARS-CoV-2 detected. This finding indicates that the presence of viral antigen is associated with IL-6-mediated inflammation, although we cannot ascertain from these experiments whether IL-6 inflammation persists in tissues in the later stages of severe COVID-19 when viral titers diminish (Bullard et al., 2020). The elevated cytokine responses seen in nasopharyngeal tissues were also detectable in the transcriptome of whole blood and isolated leukocytes from COVID-19 patients compared with the control populations available, although this analysis merits being extended to include a wider array of conditions associated with hyperinflammation (Leisman et al., 2020). Although a reduction in cytokine activity tracked clinical recovery from illness, IL-1β and IL-6 activity at the time of hospital attendance was not predictive for clinical outcome, and, in contrast to the association seen with circulating levels of IL-6 protein (Thwaites et al., 2020), we observed no clear gradient of IL-1β or IL-6 response module expression with disease severity. Our findings may help explain the recent results from randomized studies whereby neutralization of IL-6 activity by tocilizumab did not show a benefit in mortality or clinical recovery in patients with severe COVID-19 (Hermine et al., 2020; Salvarani et al., 2020; Stone et al., 2020). However, these studies did not record IL-6 activity before or after tocilizumab administration, precluding associations between cytokine activity, neutralization efficiency, and clinical outcomes. We propose that future randomized trials will need to incorporate assessments of cytokine activity in study protocols to permit mechanistic correlations between immunomodulatory interventions and disease outcomes, promoting a stratified medicine approach to host-directed therapies in COVID-19.

A consistent observation in our work was that transcriptional modules identified differences between patient groups that would not otherwise have been detected by assessment of cognate gene transcripts. An interpretation of these findings is that the downstream response to cytokine stimulation is more persistent than the expression of the cytokine gene mRNA, the stability of which is subject to trans-regulatory factors and feedback loops (Iwasaki et al., 2011; Seko et al., 2006). Moreover, transcriptional modules are intrinsically composed of genes with co-correlated expression, minimizing technical confounding of single gene measurements, demonstrated by the strongly concordant expression between the full and Nanostring subset IL-1β and IL-6 response modules. These factors may explain the discordance recorded between IL-6 gene expression and protein secretion in COVID-19 (Hadjadj et al., 2020). Moreover, cytokine levels after modulation *in vivo* do not necessarily reflect bioactivity, exemplified by the rise in IL-6 in blood following administration of tocilizumab (Nishimoto et al., 2008). We propose that cytokine response modules overcome both issues by integrating the culmination of cytokine signaling events and may be used as an *in vivo* biomonitor of cytokine activity (Hedrick et al., 2020).

### Limitations of the study

Our study has limitations. Despite drawing on four independent COVID-19 datasets, the sample sizes assessed in our study were still modest, especially for longitudinal samples, but this was limited by the data available. Assessments of the transcriptome from leukocytes and whole blood in COVID-19 may not be interchangeable and will need cross-validating, although both datasets demonstrated no association between IL-1β or IL-6 activity and severity of disease. Determining the sensitivity and specificity of the IL-1β and IL-6 response modules for their respective cognate cytokines was limited by the available datasets and the range of cytokine stimulation conditions performed in those experiments. Comparing the expression of these modules across a wider range of biologically paired cytokine stimulations will allow refinement of their accuracy. As the modules were generated from *in vitro* experiments, we sought to determine their applicability *in vivo*, assessing neutralization of cytokine activity following immunomodulation with biologic agents *in vivo*. IL-1β activity in blood and tissues was diminished days after canakinumab (Figure 3) and anakinra (Pollara et al., 2019) administration respectively, but no equivalent datasets were available to assess the IL-6 response module in the same manner. Biobanked samples from ongoing tocilizumab clinical trials in COVID-19 and other diseases may provide an opportunity to validate IL-6 module performance in this way.

### Conclusions

Our data demonstrate elevated activity of the inflammatory cytokines IL-1β and IL-6 in COVID-19 in blood and tissues and demonstrate the utility of cytokine transcriptional response modules in providing a dynamic readout of the activity of these pathways *in vivo*. We propose that use of these modules may enhance efforts to investigate the pathology of COVID-19, support development of methods to stratify patients' risk of clinical progression, and aid quantification of the biological effects of host-directed immunomodulatory therapeutics in COVID-19.

### Resource availability

#### Lead contact

Further information and requests for resources and reagents should be directed to and will be fulfilled by the Lead Contact, Dr Gabriele Pollara (g.pollara@ucl.ac.uk).

#### Materials availability

The current study made use of transcriptional modules derived from two open access publications (Jura et al., 2008; Pollara et al., 2019). The gene content for each module is available in Table S2. The R code used to calculate geometric mean expression of modules has been previously published (Pollara et al., 2017) and is freely available (https://github.com/MJMurray1/MDIScoring).

#### Data and code availability

This study did not generate new datasets or code. The title, DOI, accession number, and repository for all datasets used to support the current study are listed in Table S1.

### Data sharing

All transcriptional datasets used in this manuscript were derived from public repositories. Their source is detailed in Table S1, and the software used to analyze these data is described in the Transparent methods.

## Methods

All methods can be found in the accompanying Transparent Methods supplemental file.

## Supplemental information

Document S1. Transparent Methods, Figures S1–S3, and Tables S1 and S2
