# Supplementary material for: Transcriptional response modules characterize IL-1β and IL-6 activity in COVID-19
Source: iScience. 2020 Dec 7;24(1):101896. doi: 10.1016/j.isci.2020.101896 (PMC7721347; doi:10.1016/j.isci.2020.101896)
Supplement: Document S1. Transparent Methods, Figures S1–S3, and Tables S1 and S2 [file mmc1.pdf]

iScience, Volume *24*

Supplemental Information

Transcriptional response modules characterize IL-1β and IL-6 activity in COVID-19

Lucy C.K. Bell, Cem Meydan, Jacob Kim, Jonathan Foox, Daniel Butler, Christopher E. Mason, Sagi D. Shapira, Mahdad Noursadeghi, and Gabriele Pollara

Figure S1

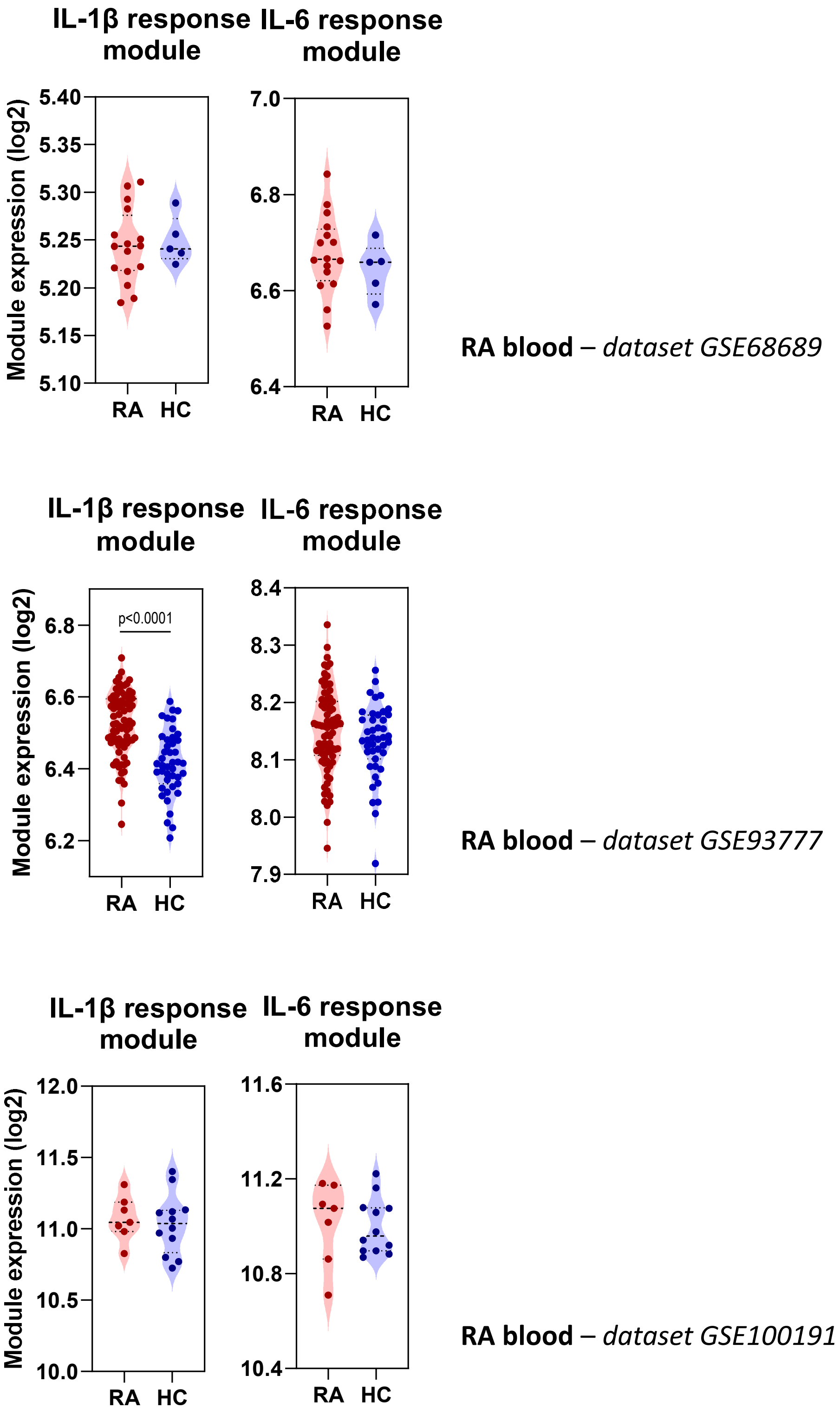

Figure S1, related to Figure 2. Cytokine response module expression in the blood of rheumatoid arthritis (RA) patients. Geometric mean expression of IL-1β and IL-6 cytokine response modules in the transcriptome of blood samples from RA patients compared to healthy controls. Statistical significance ($p<0.05$ by Mann-Whitney test) is indicated on plots. Transcriptomic datasets assessed are designated adjacent to each figure panel.

Figure S2

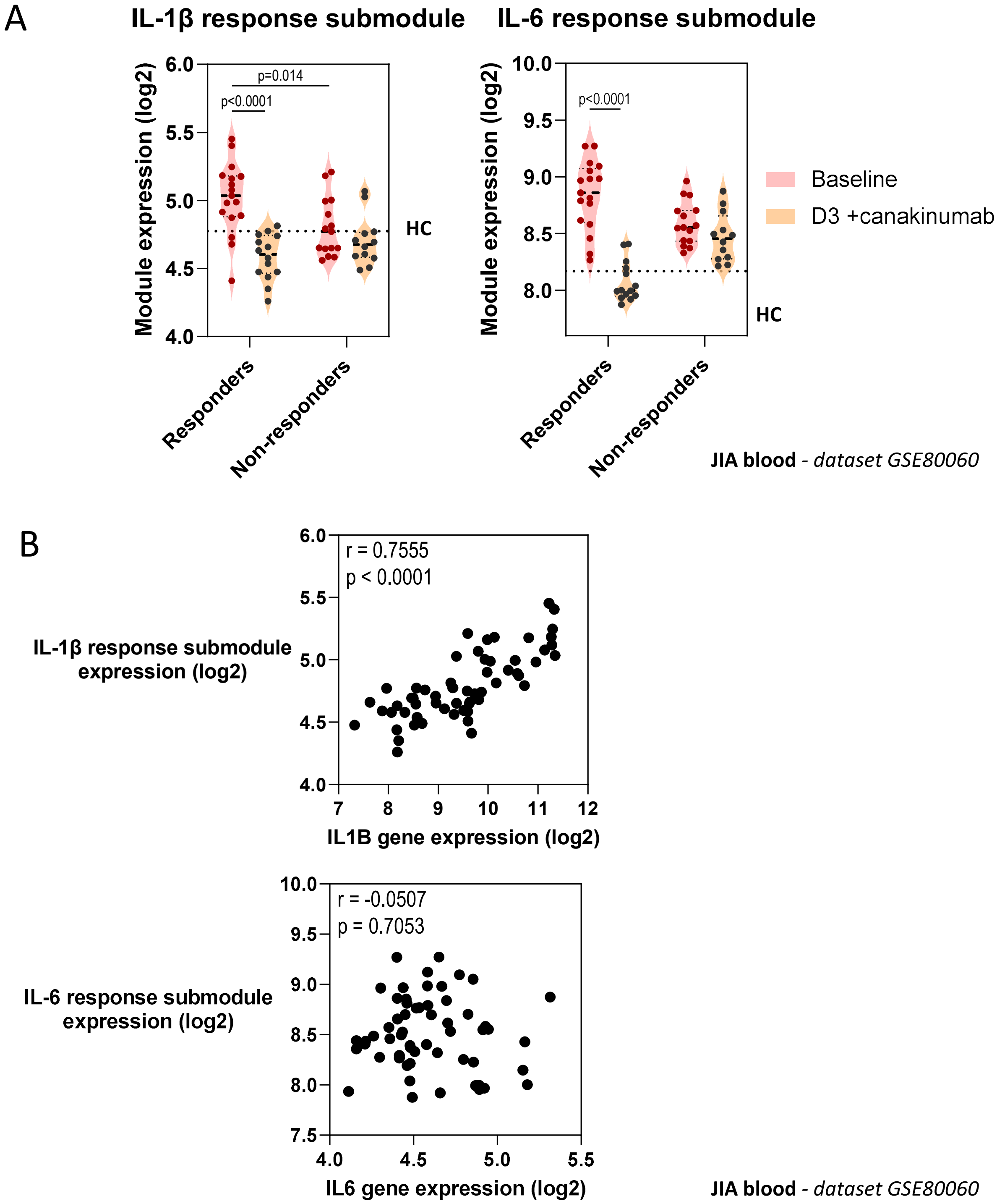

Figure S2, related for Figure 3. Effect of canakinumab on expression of cytokine genes and response submodules. A) Geometric mean expression of IL-1 and IL-6 cytokine response submodules in JIA patients before and 3 days after administration of canakinumab. Patients were subdivided into good responders (90-100% improvement) and non-responders (0-30% improvement). Dotted lines indicate median module or gene expression in healthy controls (HC) population in same dataset. Statistical significance ($p<0.05$ by Mann-Whitney test) is indicated on plots. B) Relationship between expression of cytokine response modules and cytokine genes. Statistical assessment of correlation made by Spearman Rank correlation. r = correlation coefficient. Transcriptomic dataset designated adjacent to figure panels.

Figure S3

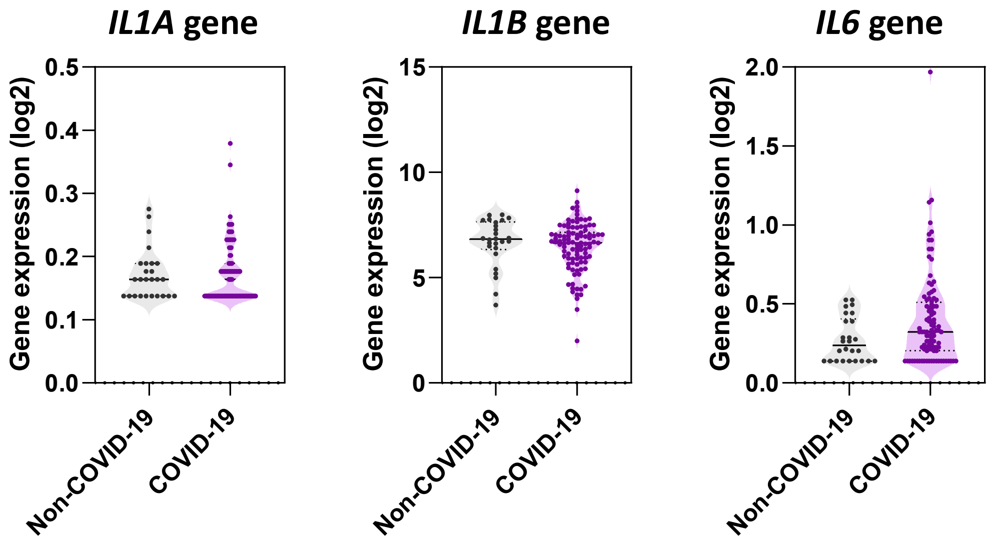

COVID-19 leucocytes - *dataset GSE157103*

Figure S3, related for Figure 6. Cytokine gene expression in leucocytes of admitted patients with and without COVID-19. Expression of *IL1A*, *IL1B* and *IL6* genes in transcriptomic profiles of blood leucocytes collected from 101 COVID-19 and 24 non-COVID-19 patients. In this study, samples were collected from patients at a median of 3.37 days from admission to hospital (Overmyer et al., 2020) . All comparisons were not significant by Mann-Whitney test. Transcriptomic dataset assessed are designated adjacent to each figure panel.

Supplemental tables

Table S1. Transcriptional datasets used in this manuscript, related to all Figures.

Title of dataset	Doi:	Accession number	Repository
Identification of IL-1 and IL-6-responsive genes in human monocyte-derived macrophages	10.1016/j.bbagrm.2008.04.006	GSE8515	NCBI GEO
Transcriptome analysis in peripheral blood mononuclear cells (PBMC) from HOIL-1-deficient patients upon TNF-α or IL-1β stimulation	10.1038/ni.2457	GSE40838	NCBI GEO
Response of HK-2 cells to stimulation with IL6 and TNF-alpha	10.1371/journal.pgen.1005734	GSE68940	NCBI GEO
Comparative gene expression in response to various inflammatory stimuli in vitro: infection-mediated versus systemic inflammation	10.1111/febs.15362	GSE126525	NCBI GEO
Gene expression data of whole blood of systemic juvenile idiopathic arthritis (SJIA) patients treated with canakinumab or placebo and age matched healthy controls	10.1186/s13075-016-1212-x	GSE80060	NCBI GEO
Gene expression from the whole blood of rheumatoid arthritis patients and normal controls.	https://doi.org/10.1016/j.cyto.2019.154960	GSE68689	NCBI GEO
Multi-omics monitoring of drug response in rheumatoid arthritis.	10.1038/s41467-018-05044-4	GSE93777	NCBI GEO
Transcriptional Signature Associated with Early Rheumatoid Arthritis and Healthy Individuals at high risk to develop the disease.	10.1371/journal.pone.0194205	GSE100191	NCBI GEO
Synovial biopsies of rheumatoid arthritis and healthy controls	10.1089/hum.2015.127	GSE77298	NCBI GEO
Transcriptomic analysis of immune response in healthy controls and COVID-19 cases using the NanoString Human Immunology Panel	10.1016/j.chom.2020.03.021	E-MTAB-8871	ArrayExpress
Large-Scale Multi-omic Analysis of COVID-19 Severity	10.1016/j.cels.2020.10.003	GSE157103	NCBI GEO
Immune complement and coagulation dysfunction in adverse outcomes of SARS-CoV-2 infection	10.1038/s41591-020-1021-2	https://covidgenes.weill.cornell.edu/	Cornell University
Impaired type I interferon activity and inflammatory responses in severe COVID-19 patients	10.1126/science.abc6027	Raw data available from corresponding author	

Table S2. Cytokine response transcriptional modules and constituent genes, related to all Figures.

Module name	Number of genes	Gene names
**IL-1β response**	57	*ADORA2A, C15ORF48, C20ORF127, C2CD4B, C7ORF63, CCL20, CCL8, CHMP1B, CSF2, CSF3, CXCL1, CXCL2, CXCL5, CXCL6, DNAJB9, EGLN1, FGF2, FOXO3, GLIS3, GNA15, HAS3, HIATL1, IER3, IL11, IL6, ITPRIP, JARID2, KCNG1, LOC100134000, LRIG1, MAP3K8, MFSD2, MGC87042, MSL3, MT1G, MT1X, MTE, MTHFD2L, NAB1, NAMPT, NFKBIZ, NR4A2, OSGIN2, PFKFB3, PIM2, RCAN1, RNF145, SERPINB4, SGK1, SLC43A3, STEAP1, STEAP2, TFAP2C, TGIF1, TWIST2, ZC3H12A, ZC3H12C*
**IL-1β response submodule**	7	*CCL20, CCL8, CSF2, CXCL1, CXCL2, IL6, NFKBIZ*
**IL-6 response**	41	*AAMP, AKIP1, ANKRD10, ARPC2, CCR1, CD14, CSDE1, CTDSP2, CTNNA1, CXADR, DOK1, GADD45B, HAMP, IDH3B, IFI16, IL16, KAT5, LDLRAP1, MAP4, MR1, MSRB2, NCF4, NDUFB8, NPC1, PGD, PI4K2A, PPARD, PSMD4, RAP1GAP, RHOC, RIN2, RNASE1, RREB1, SASH1, SDS, SP110, STIP1, TSC22D3, UBE2M, UFL1, YBX3*
**IL-6 response submodule**	7	*CCR1, CD14, HAMP, IFI16, IL16, MR1, NCF4*

Transparent methods

Datasets

All datasets used are provided in table S1. Data matrices were obtained from processed data series downloaded from the NCBI Gene Expression Omnibus (GEO) (https://www.ncbi.nlm.nih.gov/geo/) or Array Express repository (https://www.ebi.ac.uk/arrayexpress/). Probe identifiers were converted to gene symbols using platform annotations provided with each dataset. In circumstances where downloaded datasets were not $\log_2$ transformed, this was performed on the entire processed data matrix. Duplicate genes were removed after the first one identified using Microsoft Excel duplicate remover function.

IL-1β and IL-6 module derivation

We previously derived an IL-1β transcriptional module from the transcriptome of fibroblasts stimulated with IL-1β or TNFα (Pollara et al., 2019). We derived a novel IL-6 transcriptional response module from a publicly available dataset (table S1) reporting experiments of human monocyte-derived macrophages (MDM) stimulated with IL-1β (15 ng/ml) or IL-6 (25 ng/ml) for 4 hours (Jura et al., 2008). In this study the transcriptional programme of cytokine-stimulated MDM was assessed by microarrays and hierarchical clustering was performed using Euclidean distance and average linkage method. This approach identified several unique clusters of genes differentially expressed following stimulation with each cytokine. Genes in clusters D & E showed elevated expression following stimulation by IL-6, but not by IL-1β. We combined the list of genes within these two clusters, removed duplicate or non-annotated genes, and termed this the IL-6 response module.

We applied the above IL-1β and IL-6 response modules to two studies where transcriptional profiling was performed using the Nanostring nCounter Human Immunology_v2 panel, which assesses the expression of a subset of the whole genome (579 genes) (Hadjadj et al., 2020; Ong et al., 2020). Consequently, only a subset of the modules' constituent genes was present in these datasets (table S2). To verify the validity of applying our method to these datasets, we generated new cytokine response submodules using only genes from this subset, and showed them to provide the same discrimination of IL-1β and IL-6 responses as the parent modules (fig S2).

Module expression assessment

The expression of transcriptional modules was derived by calculating the geometric mean expression of all constituent genes, as previously described (Pollara et al., 2017). The scripts used allowed the absence of a constituent gene in the analysed dataset, a scenario that did not affect geometric mean calculation. Gene set enrichment analysis was also used for modular expression assessment in nasopharyngeal samples, as previously described (Ramlall et al., 2020; Subramanian et al., 2005).

Statistical analysis

All module score calculations were calculated in R v3.6.1 and RStudio v1.2.1335, using scripts generated and deposited in our previous publication (https://github.com/MJMurray1/MDIScoring) (Pollara et al., 2017). Mann-Whitney tests, Spearman rank correlations and Kruskal-Wallis tests were calculated in GraphPad Prism v8.4. Kruskal-Wallis testing was chosen to determine the presence of variability in the expression of cytokine response modules or cytokine gene over time since hospital admission (fig 5A) or between different categories of COVID-19 disease severity (fig 5B). This non-parametric test was chosen as we could not assume the expression of these variables was Normally distributed. In fig 5A patient samples were aligned according to days from hospital admission, and then binned into day interval categories (4-6, 7-9, 10-12 and 12+ days following admission), yielding 4, 7, 8 and 3 samples in each group. Kruskal-Wallis testing was performed on these binned categories to identify variation in the expression of modules or genes between these categories, with the Bonferroni method used for multiple testing correction.

Role of funders

The funding sources played no role in conceiving the study, performing data analyses, preparing the manuscript or deciding to submit it for publication.

Ethics statement

The manuscript makes use of publicly available datasets, the use of which required no further ethical approval.

Supplemental references

Boisson, B., Laplantine, E., Prando, C., Giliani, S., Israelsson, E., Xu, Z., Abhyankar, A., Israël, L., Trevejo-Nunez, G., Bogunovic, D., et al. (2012). Immunodeficiency, autoinflammation and amylopectinosis in humans with inherited HOIL-1 and LUBAC deficiency. Nat. Immunol. *13*, 1178–1186.

Brachat, A.H., Grom, A.A., Wulffraat, N., Brunner, H.I., Quartier, P., Brik, R., McCann, L., Ozdogan, H., Rutkowska-Sak, L., Schneider, R., et al. (2017). Early changes in gene expression and inflammatory proteins in systemic juvenile idiopathic arthritis patients on canakinumab therapy. Arthritis Res. Ther. *19*, 13.

Broeren, M.G.A., de Vries, M., Bennink, M.B., Arntz, O.J., Blom, A.B., Koenders, M.I., van Lent, P.L.E.M., van der Kraan, P.M., van den Berg, W.B., and van de Loo, F.A.J. (2016). Disease-Regulated Gene Therapy with Anti-Inflammatory Interleukin-10 Under the Control of the CXCL10 Promoter for the Treatment of Rheumatoid Arthritis. Hum. Gene Ther. *27*, 244–254.

Das, A.S., Basu, A., Kumar, R., Borah, P.K., Bakshi, S., Sharma, M., Duary, R.K., Ray, P.S., and Mukhopadhyay, R. (2020). Post-transcriptional regulation of C-C motif chemokine ligand 2 expression by ribosomal protein L22 during LPS-mediated inflammation. FEBS J.

Hadjadj, J., Yatim, N., Barnabei, L., Corneau, A., Boussier, J., Smith, N., Péré, H., Charbit, B., Bondet, V., Chenevier-Gobeaux, C., et al. (2020). Impaired type I interferon activity and inflammatory responses in severe COVID-19 patients. Science (80-.). *369,* 718–724.

Jura, J., Wegrzyn, P., Korostyński, M., Guzik, K., Oczko-Wojciechowska, M., Jarzab, M., Kowalska, M., Piechota, M., Przewłocki, R., and Koj, A. (2008). Identification of interleukin-1 and interleukin-6-responsive genes in human monocyte-derived macrophages using microarrays. Biochim. Biophys. Acta *1779,* 383–389.

O'Brown, Z.K., Van Nostrand, E.L., Higgins, J.P., and Kim, S.K. (2015). The Inflammatory Transcription Factors NFκB, STAT1 and STAT3 Drive Age-Associated Transcriptional Changes in the Human Kidney. PLoS Genet. *11*, e1005734.

Ong, E.Z., Chan, Y.F.Z., Leong, W.Y., Lee, N.M.Y., Kalimuddin, S., Haja Mohideen, S.M., Chan, K.S., Tan, A.T., Bertoletti, A., Ooi, E.E., et al. (2020). A Dynamic Immune Response Shapes COVID-19 Progression. Cell Host Microbe *27,* 879–882.e2.

Overmyer, K.A., Shishkova, E., Miller, I.J., Balnis, J., Bernstein, M.N., Peters-Clarke, T.M., Meyer, J.G., Quan, Q., Muehlbauer, L.K., Trujillo, E.A., et al. (2020). Large-Scale Multi-omic Analysis of COVID-19 Severity. Cell Syst.

Pollara, G., Murray, M.J., Heather, J.M., Byng-Maddick, R., Guppy, N., Ellis, M., Turner, C.T., Chain, B.M., and Noursadeghi, M. (2017). Validation of immune cell modules in multicellular transcriptomic data. PLoS One *12*, e0169271.

Pollara, G., Turner, C.T., Tomlinson, G.S., Bell, L.C., Khan, A., Peralta, L.F., Folino, A., Akarca, A., Venturini, C., Baker, T., et al. (2019). Exaggerated in vivo IL-17 responses discriminate recall responses in active TB. BioRxiv.

Ramlall, V., Thangaraj, P.M., Meydan, C., Foox, J., Butler, D., Kim, J., May, B., De Freitas, J.K., Glicksberg, B.S., Mason, C.E., et al. (2020). Immune complement and coagulation dysfunction in adverse outcomes of SARS-CoV-2 infection. Nat. Med. *26,* 1609–1615.

Subramanian, A., Tamayo, P., Mootha, V.K., Mukherjee, S., Ebert, B.L., Gillette, M.A., Paulovich, A., Pomeroy, S.L., Golub, T.R., Lander, E.S., et al. (2005). Gene set enrichment analysis: a knowledge-based approach for interpreting genome-wide expression profiles. Proc. Natl. Acad. Sci. USA *102,* 15545–15550.
